# The influence of pain on community reintegration after spinal cord injury

**DOI:** 10.1111/papr.13439

**Published:** 2024-11-19

**Authors:** Valerie Henderson, Mokgadi Kholofelo Mashola

**Affiliations:** ^1^ Department of Physiotherapy, School of Therapeutic Sciences, Faculty of Health Sciences University of the Witwatersrand Johannesburg South Africa; ^2^ Department of Physiotherapy, School of Healthcare Sciences, Faculty of Health Sciences University of Pretoria Pretoria South Africa

**Keywords:** neuropathic pain, pain severity, shoulder pain, WUSPI

## Abstract

**Background:**

Community reintegration is an important goal for people living with a spinal cord injury (SCI), and pain is suspected to limit reintegration due to its limitations in daily functioning, mood, and sleep.

**Objectives:**

To determine the influence of pain on community reintegration in manual wheelchair users with SCI.

**Methods:**

The Reintegration to Normal Living Index was used to determine community reintegration, while the DN4 and the Wheelchair User's Shoulder Pain Index were used to determine the presence of neuropathic and shoulder pain respectively. Associations and differences between the pain variables and participants with and without pain were analyzed with Spearman correlations and Mann–Whitney *U*‐tests using SPSS v27 at 0.05 significance level and 95% confidence interval.

**Results:**

Of the 122 participants, 85.2% reported current pain, with a 77.7% median for community reintegration. Neuropathic pain (53.3%) was more common and severe than nociceptive shoulder pain (14.8%). There was no significant difference in community reintegration between participants with and without pain, nor any correlation between the overall presence of pain and community reintegration. The severity of pain, particularly shoulder pain, was negatively associated with taking trips out of town (*p* < 0.01), and overall community reintegration (*p* < 0.05).

**Conclusion:**

It is not the mere presence of pain that influences community reintegration, but rather the severity and the location of pain. Shoulder care and pain management need to be included in the rehabilitation program, as these are important considerations when rehabilitating people with SCI back into their communities.

## INTRODUCTION

A spinal cord injury (SCI) causes a devastating loss with many changes in the lives of the affected individuals. Not only are there changes or complete loss in motor and sensory function, but people with SCI (PWSCI) are faced with many secondary health conditions (SHCs) such as pain, pressure ulcers, and urinary tract infections.^1^ Secondary health conditions often limit the rehabilitation progress PWSCI have made, or make their usual functioning more difficult, affecting activities of daily living, mobility and work, their mental health, personal behavior, and quality of life (QOL).^2^ Pain is one of the most problematic SHCs and affects 70% of PWSCI, with one‐third experiencing severe, disabling pain.^3^, ^4^, ^5^, ^6^ Pain can develop shortly after a SCI or years later, with chronic pain associated with depression, poor sleep, and poor health.^7^

Pain after SCI may present as neuropathic, nociceptive, or visceral.^8^ Neuropathic pain is due to damage to the nervous system; it can present above, at, or below the level of injury and can be spontaneous at rest, or stimulus provoked. Burke et al.^8^ found that PWSCI with neuropathic pain reported more pain days, more severe pain, and more time seeking medical assistance than those with nociceptive pain. Neuropathic pain was also reported to affect mood, sleep, and day‐to‐day activities more than nociceptive pain. Neuropathic pain can be difficult to manage, with limited evidence regarding the efficacy of the medication and long‐term effects; pain relief is limited, and high pain levels persist even with pharmaceutical intervention.^4^, ^8^, ^9^, ^10^

It is of particular concern for PWSCI if pain relief is not achieved due to the reported interferences with daily activities,^8^ transfers, and wheelchair dexterity.^11^ The ability to successfully self‐propel a manual wheelchair can assist a person's ability to participate in community activities. Cooper et al.^12^ found that manual wheelchair users (MWU) were able to mobilize with an increased speed and increased frequency which had a positive correlation with community participation, compared to power wheelchair users. However, the continuous use of a manual wheelchair may result in the possibility of developing an upper body repetitive strain injury. The shoulder is the most common site of injury and pain in MWU, with a reported incidence of 32%–78% due to increased forces around the shoulder during pressure relief, incline propulsion, and at the start of a push.^11^ Mozingo et al.^13^ found that shoulder impingement risk was highest during wheelchair propulsion and scaption, which is when one lifts their arms forward from the sides of the body, at a slight angle. However, in both studies, it is important to note that the frequency of these tasks performed in everyday life is far more than what was done and measured in these studies. Therefore, although using a manual wheelchair provides the freedom of movement and the potential to be better integrated into the community, all these high‐demand tasks can put MWU at risk for injury and shoulder pain.

Community reintegration and participation have been described as the ultimate goal for people with disability.^14^ Regaining life roles, maintaining relationships, and finding a sense of purpose are vital for integration back into the community, and are related to life satisfaction and QOL. Participation in activities outside of the home allows for these important connections.^15^ However, there are multiple barriers to community reintegration such as environmental, social, infrastructure, employment, and economic barriers as well as mobility aids/equipment barriers.^15^, ^16^ With all these barriers influencing community reintegration, especially in low‐income countries,^17^, ^18^ community reintegration is often a challenge for many in the disabled population.^19^

After observation of both pain and community reintegration literature, it can be hypothesized that pain, in some way, may influence community reintegration.^20^ Although pain was not the most frequently occurring barrier to community reintegration one‐year post‐discharge from acute rehabilitation, pain prevalence progressively increased during the year.^21^ This could potentially continue over time and ultimately affect community reintegration. Similarly, Bangladeshi participants rated health‐related problems as the fifth most common barrier to their community reintegration, with pain and bowel and bladder function being the most common health‐related barriers.^16^ Pain is a common secondary complication of SCI, and for a variety of reasons such as finding the best medication combination, with fewer side effects, and cost‐effectiveness, pain is difficult to manage.^22^ Due to different cultures and the subjective nature of pain, as well as varied opportunities in high‐income countries versus low‐income countries, it will be beneficial to determine the impact of pain on community reintegration in the South African context. Understanding how pain affects community reintegration may influence how we treat pain in PWSCI in South Africa and ultimately influence community reintegration and QOL. This study therefore aimed to determine the influence of pain on community reintegration of manual wheelchair users with SCI, especially with regards to associations between neuropathic pain, nociceptive pain, and severity of pain and community reintegration.

## RESEARCH METHODS

A correlational design was used to investigate pain and how it influenced not only disability but also functioning in PWSCI who use manual wheelchairs.^23^ The data collected included pain information (presence of and medication use), wheelchair function, QOL, community reintegration, as well as scapular dyskinesis and pectoralis minor muscle length. This paper reports only on the influence of pain on community reintegration. The primary data was collected from February 2019 to March 2020 and the research procedures were conducted as set out in the published protocol.^23^

### Setting

The study setting included the homes of participants who were rehabilitated in four public and one private rehabilitation facility in Gauteng. The participants were only included if they lived within a 500 km radius of their discharging hospitals, with all participants residing either in Gauteng, Limpopo, Northwest, or Mpumalanga. Due to the nature of the study (face‐to‐face in the community), potential participants residing beyond the 500 km driving distance were excluded from the study.

### Study population and sampling strategy

The sample size was determined using the events per variable approach^24^ for the overall study.^23^ The variables included were age, gender, occupation, duration of SCI, neurological level of injury, completeness of injury, pectoralis minor length, and scapular dyskinesis. Using this calculation, EPV > 5, ie number of events >5 × 8 = 40, therefore sample size equals 40/0.35, ie at least 115 participants were required.

*Inclusion Criteria*

- All adults with T2 and below paraplegia irrespective of etiology, classification, and completeness of injury.
- Six months post‐discharge to allow for optimum levels of independence and for soft tissue changes in the shoulder to occur.

*Exclusion Criteria*

- Patients who were re‐admitted due to any SHCs during the time of the primary data collection.

### Data collection

A sociodemographic capture tool was used to determine the participant's demographic, injury profile, and pain characteristics. The Numeric Rating Scale (NRS) was used to determine the severity of the pain. Higher numeric scores indicate greater pain intensity, with the NRS being valid and recommended as the most appropriate outcome measure for pain intensity post‐SCI.^25^

The RNLI questionnaire was used to assess the degree to which participants perceived to be reintegrated into normal social activities. The RNLI has 11 items covering aspects of mobility, self‐care, daily activity, recreational activity, and family roles.^26^ Each question is scored on a numeric scale with statements, from zero to 10, with zero representing no reintegration and 10 representing complete reintegration. The total score is then converted to a percentage score to determine the level of perceived integration into the community. The RNLI is a reliable and valid measure for community reintegration for people living with SCI and is validated in the South African context.^27^

The DN4 questionnaire was used to determine whether participants experienced neuropathic pain or not. It is a 10‐item questionnaire totaling a score out of 10. Seven questions are related to the type of pain, and three questions are related to a clinical exam. Participants are required to give a “yes/no” response, and the cutoff value to diagnose neuropathic pain is a minimum of four out of ten.^28^ Although the DN4 has been validated and can diagnose neuropathic pain with high accuracy,^29^ its psychometric properties have not yet been tested in South Africa.

The WUSPI measures shoulder pain in wheelchair users during functional activities, including transfers, wheelchair mobility, self‐care, and general activities. The 15 items are rated on a numeric scale from zero to 10. To accommodate for items that were not applicable, the WUSPI Performance corrected score was calculated, where the total score was divided by the number of items that were performed, multiplied by 15.^30^ The WUSPI has been proven to be reliable and valid for people with SCI^31^ and is yet to be validated for the South African context.

### Data analysis

Data analysis was performed using SPSS version 28. Descriptive data was analyzed for participants' demographic and injury profiles. A test for normality was completed of the RNLI percentage scores, which showed that data was not normally distributed. Therefore, non‐parametric testing was used to meet the objectives. Mann–Whitney *U*‐tests were used to determine the differences between groups, and Spearman correlations and Linear regression tests were used to report associations and the influence of pain on community reintegration. All testing was done at the 0.05 level of significance and 95% confidence intervals. All correlations reported followed the guidelines by Safrit and Wood (1995) (cited in^32^):

- No correlation: *r* = 0–0.19
- Low correlation: *r* = 0.2–0.39
- Moderate correlation: *r* = 0.4–0.59
- Moderately high correlation: *r* = 0.6–0.79
- High correlation *r* = ≥ 0.8

## ETHICAL CONSIDERATIONS

Unconditional ethical permission from the Human Research Ethics Committee of the University of Witwatersrand was received (approval number M210516) for this study, as well as the Faculty of Health Science Research Ethics Committee of the University of Pretoria (approval number 125/2018). Hospital permissions from the relevant hospitals were also obtained to access their databases. All participants received an information leaflet which was explained to them in a language they could understand, and they provided written informed consent to participate in the study. All data, including the consent, are stored for safekeeping at the Physiotherapy Department of the University of Pretoria. Participants were not penalized or prejudiced for leaving the study at any time and no cost to the participants was incurred.

## RESULTS

### Sociodemographic and injury profile

A total of 122 participants were included in the study with the majority being male (*n* = 83, 68%) and Black (*n* = 104, 85.2%). The mean age of participants was 39.7 years (SD 11.1) and the mean age of injury was 32.60 years (SD 10.7). Sixty‐two (50.8%) participants have been living with their SCI for 1–5 years, and 34 (27.9%) for 6–10 years. Ninety‐one (74.6%) participants are currently aged between 31 and 60 years, with a mean age of 39.70, which is the range of a working population. One hundred and four injuries were traumatic and 18 non‐traumatic. The most common cause of traumatic injuries was motor vehicle accidents, (41%), followed by gunshot injuries (26%). Complete SCI was more common than incomplete SCI (*n* = 93, 76.2%). The most common neurological level of injury was T6‐T12, accounting for 73.8%, with levels T6 as well as T12 being the most reported NLI, both *n* = 25 (20.5%). However, a total of 59% were unemployed. Fifty‐six (45.9%) of the participants lived in the township area, and *n* = 62 (50.8%) stayed with their own families.

### Pain presentation

Most of the participants, 85.2%, reported current pain with one participant (0.8%) reporting up to five simultaneous painful areas. Most participants had one painful area (48.4%). The different pain locations were expressed in order of severity, with P1 being the most severe and P5 the least severe pain experience. The severity of P1 was at a mean of 6.7 (SD 2.25) using the NRS. The DN4 questionnaire confirmed that 65 participants (53.3%) reported overall neuropathic pain with P1 located in the lower limbs below the level of injury (*n* = 41, 39.4%). Participants mostly described this neuropathic pain as burning (*n* = 61, 93.8%) and pins‐and‐needles (*n* = 54, 83.1%). Other than neuropathic pain, the most common site for nociceptive pain was the back at the injury site (*n* = 11, 9%), and the upper back above the injury site (*n* = 10, 8.2%) for P1. According to the descriptions of pain behavior and pain location subjectively described by participants, neuropathic pain was the most common type of pain experienced in P1 (*n* = 75, 72.1%) and P2 (*n* = 29, 27.9%), whereas nociceptive pain was more common in P3 (*n* = 7, 6.7%) and P4 (*n* = 3, 2.9%). Only 18 (14.8%) of the participants overall reported shoulder pain when asked if they had shoulder pain or not. The non‐dominant shoulder accounted for the most pain, *n* = 8 (44.4%), bilateral shoulders *n* = 6 (33.3%), and the least pain occurring in the dominant shoulder *n* = 4 (22.2%). Of these 18 participants, 5 had shoulder operations for various reasons such as ORIF post shoulder dislocation, rotator cuff repair, or ORIF of the humeral head and humeral shaft due to a gunshot wound and fracture respectively. With regard to participants who experienced shoulder pain (*n* = 18, 14.7%), total scores for the WUSPI ranged from 3/150–129/150 (Median = 57.0, IQR = 62.0) and the WUSPI showed that shoulder pain was the most intense (Median = 6.0) during tub or shower transfers (IQR = 4.75); prolonged wheelchair propulsion and up inclines (IQR = 3.875 and 6.0 respectively); lifting objects from an overhead shelf (IQR = 5); and while sleeping (IQR = 8.25) (Table 1).

**TABLE 1:** Descriptive statistics of the WUSPI.

WUSPI activity, *n = 18*	Median	IQR
Transfer from bed to WC	4.5	5.25
Transfer from wheelchair to car	4.5	7.25
Transfer from wheelchair to tub or shower	6.0	4.75
Loading your wheelchair into car	4.5	3.25
Pushing your wheelchair for 10 min or more	6.0	3.875
Pushing up ramps or inclines outdoors	6.0	6.0
Lifting objects down from an overhead shelf	6.0	5.0
Putting on pants	0	6.25
Putting on t‐shirt or pullover	0	6.50
Putting on a button‐down shirt	0	2.0
Washing your back	5.0	3.0
Usual daily activities at work or school	6.0	6.25
Driving	2.5	6.75
Performing household chores	4.5	5.25
Sleeping	6.0	8.25
WUSPI total	57.0	62.0
WUSPI performance corrected score	61.5	74.0

### Community reintegration

**FIGURE 1:**
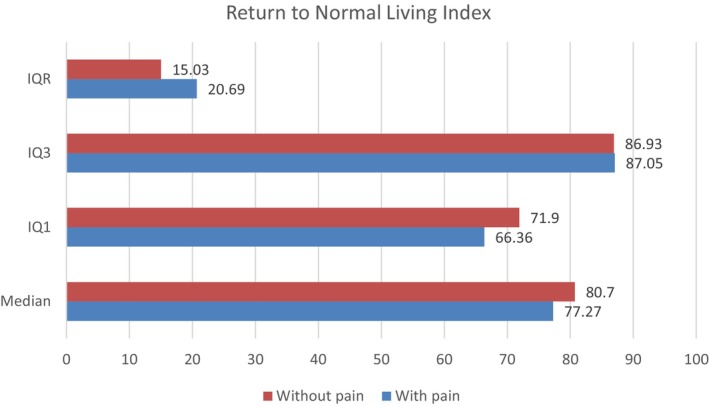
RNLI overall percentage descriptive statistics, comparing those with pain (*N* = 104) and those without pain (*N* = 18).

Total RNLI scores ranged from 41.5/110 to 110/110, with a median of 85.5 (IQR 21.88). The poorest area of integration was moving around the community as necessary, with a median of 7 (IQR 4) (Table 2). There were insignificant differences in the participants' reintegration scores when comparing those with pain (median = 77.27%, IQR = 20.69) to those without pain (median = 80.7%, IQR = 15.03) (Figure 1). The RNLI item *“I move around my living quarters as necessary*” was the only significant item, however, it was participants with pain that scored higher than those without pain. All other items were not significant, suggesting that the mere presence of pain does not influence community reintegration.

**TABLE 2:** RNLI descriptive statistics for the total sample and individuals with and without pain (*N* = 122).

	Total sample (*n* = 122)		With pain (*n* = 104)		Without pain (*n* = 18)		*p*‐value
Reintegration to normal living index item	Median	IQR	Median	IQR	Median	IQR	
I move around my living quarters as necessary	8.5	3.0	9.0	3.0	8.0	1.63	* 0.040
I move around my community as necessary	7.0	4.0	7.0	4.0	7.0	3.25	0.892
I am able to take trips out of town as necessary	8.0	5.0	8.0	5.0	7.250	3.13	0.431
I am comfortable with how my self‐care needs are met	9.0	3.0	9.0	3.0	8.5	4.0	0.751
I spend most of my day occupied in a work activity that is necessary/important to me	8.0	4.0	8.0	4.0	9.0	2.0	0.075
I am able to participate in recreational activities as I want to	9.0	4.0	9.0	4.0	8.5	3.25	0.731
I participate in social activities as necessary/desirable to me	8.0	4.0	8.0	4.75	9.0	3.25	0.399
I assume a role in my family which meets my needs and those of other family members	8.0	5.0	8.0	5.0	9.0	4.13	0.208
In general, I am comfortable with my relationships	8.0	5.0	8.0	5.0	9.0	4.25	0.435
In general, I am comfortable with myself when I am in company of others	9.0	3.0	9.0	3.0	10	2.0	0.179
I feel I can deal with life events as they happen	8.25	3.0	8.0	3.0	9.0	2.0	0.287
RNLI overall percentage	77.73	19.88	77.27	20.682	80.68	15.0	0.516

* Correlation is significant at the 0.05 level (2‐tailed).

### Influence of overall pain on community reintegration

There were no statistically significant differences between the RNLI scores of those with and without pain (*U* = 846.0, *p* = 0.516). Mann–Whitney test results were also not statistically significant between participants with neuropathic pain versus those without, and for participants with shoulder pain versus those without, on the RNLI score (*U* = 1494.50, *p* = 0.066 and *U* = 861.50, *p* = 0.591) respectively. Furthermore, completeness of injury of those with pain did not influence community reintegration (*U* = 952.5, *p* = 0.869).

### Nociceptive shoulder pain and community reintegration

A Spearman correlation between WUSPI performance corrected score and RNLI percentage showed no significant association between shoulder pain and RNLI (*r* = −0.39, *p* = 0.115). However, the RNLI item “*able to take trips out of town*” showed a high negative correlation with WUSPI item “*experiencing pain while loading wheelchair into the car*” (*r* = −0.821, *p* = 0.045), and a moderate negative correlation with WUSPI item “*pushing up ramps and inclines outdoors*” (*r* = −0.546, *p* = 0.02).

### Neuropathic pain and community reintegration

A simple linear regression was used to test if having neuropathic pain influenced community reintegration. Although the p‐value was significant, there was no correlation between neuropathic pain and community reintegration (*r* = 0.19, *p* = 0.018), with a low predictor *p*‐value of having pain resulting in lower scores of community reintegration (*β = −0.191, p =* 0.036). Furthermore, linear regression tests were done with neuropathic pain and all aspects of the RNLI. Although there was a trend towards less community reintegration with neuropathic pain, no sub‐sections of the RNLI yielded any significant correlations.

### Influence of severity of pain on community reintegration

Spearman correlation tests revealed P2 severity (identified as mostly neuropathic pain) to have a low negative correlation with moving around the community (*r* = −0.208, *p* = 0.022). P3 severity (identified mostly as shoulder pain) showed a low negative correlation with taking trips out of town, (*r* = −0.273, *p =* 0.002), and a low negative correlation with overall community reintegration, RNLI percentage (*r* = − 0.224, *p =* 0.013). These findings suggest that both shoulder and neuropathic pain severity is associated with community reintegration.

Bivariate linear regression tests were conducted to confirm if neuropathic pain as the most common pain type of pain in P1, would predict reduced reintegration in the community. There was a negative and low correlation between the most severe pain (P1) and community reintegration (*r* = −0.153, *p*‐value = 0.046), suggesting that as pain severity increases, then community reintegration is negatively affected. However, pain severity was not found to predict community reintegration (*β* = −0.686, *p* = 0.092), suggesting that although correlated, there may be factors other than pain, that predict community reintegration.

## DISCUSSION

This sample of traumatic causes of injury, particularly in males, is common. Pilusa et al.,^2^ who conducted their study in Gauteng, support this finding with traumatic causes (82.4%) resulting in SCI in males (82.4%). However,^33^ found more non‐traumatic causes (54%) in women in KZN, which was attributed to the high rate of HIV infection in this area. Epidemiological data also varies in different countries, for example, causes of traumatic injuries are becoming more prominent in the elderly with falls in high‐income countries,^34^ while exposure to assault accounts for many younger aged traumatic injuries in South Africa.^35^ The young middle‐aged group of participants with SCI is similar to other local studies,^2^, ^36^ reporting means ages of 44.5 and 41 years, respectively, as well as findings in other low‐income countries such as Brazil,^37^ compared to higher mean ages (51 years) in high‐income countries such as United States and Australia.^37^ The high reports of pain reported in this study, including neuropathic pain being the most common and most severe type of pain, were unsurprising and are consistent with other findings.^8^, ^38^ This pain rating falls into the moderate pain category, 4–7, with severe pain ratings being 8–10, and shows that pain is an important secondary health condition to consider.

Shoulder pain prevalence in this study was low compared to previous literature; 14.8% versus 73.3%^39^ and 55%.^40^ A possibility for this may be that only participants with paraplegia were included in this study. It is thought that patients with tetraplegia experience more shoulder pain due to not only the musculoskeletal impact on the shoulders, but due to the combination of weakness and neurological fallout as well.^40^ Furthermore, the likelihood of having shoulder pain increases with contracture and spasticity,^41^ and reduced ROM,^40^ which is more likely to occur in those with tetraplegia than paraplegia. Haubert et al.^42^ found that there was less shoulder pain onset compared to the control group, when a prevention program that included movement optimization was performed. It is plausible that participants in this study may have used shoulder pain prevention strategies or followed a shoulder exercise program that decreased the likelihood of shoulder pain. The low prevalence of shoulder pain reported by participants may have been a reason why no correlation between shoulder pain and community reintegration was found. These findings mirror those by Gutierrez et al.,^43^ however in their study, the WUSPI was correlated with the Communities Activity checklist, which has more social items compared to mobility items, and this may have contributed to the reason for their no correlation.

The most intense shoulder pain in this study was felt with pushing up an incline, lifting objects from an overhead shelf, and transferring into a bath. Both pushing up an incline and lifting arms overhead are activities that can be performed in the community and pushing up an incline is consistent with literature for times of increased shoulder pain.^11^, ^13^ Ramps provide accessibility to PWSCI using manual wheelchairs^19^; however, it is the finding of pain during this activity that limits accessibility; as PWSCI may avoid ramps to avoid pain, thereby limiting themselves to what areas they can access. Furthermore, whether transferring into a privately owned car or using public transport, car transfers are one of the most challenging transfers, and the need to load a wheelchair further increases strain on the upper limbs and shoulders.^44^ The ability to use transport has been found to increase overall community reintegration as well as increase the odds of being employed.^45^ One must be able to travel by motor vehicle to access the community and potential workplaces, especially to areas at a further distance. If a PWSCI is using a car daily and transferring independently as well as loading his/her wheelchair, one must consider the potential for pain in the shoulders.^44^

Although the age findings in this current study depict a possible working population age, most of the participants were unemployed, and this is most likely due to not only high unemployment rates in South Africa, but also the difficulties experienced in employment opportunities for people with a SCI.^33^ In contrast, Buys et al.^36^ found that 80.5% of their participants were employed post‐SCI. Potential reasons for this contrast with findings by Buys et al.^36^ may be due to their participants having incomplete injuries, perhaps making accessibility to work easier or that the majority of participants lived in urban areas. In urban areas, employment rates are generally higher, and access to transport is more readily available; with not only the physical environment being more conducive to wheelchairs and the distance to travel to use potential transport less than township areas, but also the availability to use various modes of transport. The authors had previously found (in the same sample population) that participants with a complete injury experienced higher pain severity by 1.27 points than those with incomplete SCI.^46^ However, completeness of injury on its own does not influence community reintegration in our current study. The authors only included people with paraplegia, therefore, these findings may differ when people with tetraplegia are considered.

The majority of the participants resided in township areas, which in South Africa means often informal living and over‐crowding.^47^ The environment is sandy, uneven, and more than likely not wheelchair user friendly. The median score of the RNLI (77.73%) falls into the category of moderate restriction in participation.^48^ Although Buys et al.^36^ also found moderate restrictions in participation, their RNLI score was 9% lower at a mean score of 68%.^36^ This difference was found despite both studies having similar demographics in terms of gender, age, and participants living in both rural and urban environments. Buys et al.^36^ included participants with tetraplegia, which may have influenced their levels of reintegration, due to the nature of the severity of the injury with less mobility and function. Interestingly, Buys et al.^36^ found higher levels of productive activity resulted in higher scores on the RNLI, whereas a majority of our participants were not employed, yet still had higher scores on the RNLI. Buys et al.^36^ included participants who were discharged 12 months before the start of their study, however, there is no information regarding the time post‐SCI of participants in their study. Comparatively, half of the participants in this study have been living with their SCI for more than 5 years. It may be possible that the longer PWSCI live with their injury, the better their integration into their community is. This may be due to physical improvements in independence and mobility^49^ or due to PWSCI perceiving their integration to be better the longer they live with their injury.^50^ For example, Nizeyimana et al.^51^ found the mean score of perceived community reintegration to be significantly higher in PWSCI who lived with their injury for 11–15 years, compared to those who only lived with their injury for 1–5 years.

There are no normative values of the RNLI, but the higher percentage in this study may indicate that participants perceived their integration towards the higher end. There is an adjustment process that takes place post‐SCI, and perhaps, the idea of participation to PWSCI prior to injury may have shifted after the injury. There are many facilitators to community reintegration, such as overall health stability and social support.^50^, ^52^ 80.3% of the participants in this study reported no comorbidities, and most participants lived with their families, which may explain the high RNLI in this study. Those who experienced pain scored lower on the RNLI than those who did not have pain, but overall, the results were not significant. Contrastingly, Buys et al.^36^ found that the presence of pain was significantly related to lower scores on the RNLI. They did not specify what type of pain participants had, nor the severity. Their sample size was 41 compared to the current sample size of 122, and they included participants with tetraplegia who accounted for 36.6% of the sample size, potentially accounting for the difference in results.

Noreau et al.^53^ found that the perceived limitation of neuropathic pain on participation was more important than experiencing pain. Some activities were still performed despite pain, and this may explain why this study did not find any association between neuropathic pain and community reintegration. There may be various other factors that contribute to the level of community reintegration with the presence of pain, or perhaps PWSCI can maintain some level of community reintegration despite their neuropathic pain levels. This may potentially be due to their self‐efficacy^51^ or the cultural behaviors of South Africans, where there are high levels of resilience to adversity.^54^ For example, Nizeyimana et al.^51^ found a positive correlation between perceived community reintegration and general self‐efficacy and that the social functioning factor of the self‐efficacy scale predicted the highest variance of perceived community reintegration. We do not know the participants' self‐regulation, levels of motivation, or confidence that may explain our findings. To then consider the cultural behavior of South Africans, this study's findings may expand to the nature of South Africans dealing with the hardships of living in townships, where 45.9% of this study's population reside. Resilience in South Africans comes mostly from self, with some family support and little social‐ecological support.^54^ In this study, half of the participants lived with immediate family and the influence of resilience and family support on community reintegration may be a potential research area for further pursuit.

Our findings indicate that the more severe the pain, the less the integration in the community, particularly in moving around the community. Donnelly and Eng.^55^ also found that pain intensity was correlated to community reintegration at 6 months after injury and pain intensity accounted for 25% of the variance in RNLI scores. Types of pain and pain location were not identified, therefore the finding in our study further adds to our clinical knowledge. Individuals with lower levels of pain report interference with psychological effects like mood, sleep, and life enjoyment more frequently, whereas individuals with higher pain levels more frequently report interference in social relationships and daily activities.^56^ Kuzu et al.^57^ found that within‐person fluctuations in day‐to‐day pain showed no decrease in same‐day social participation, but between‐person comparison showed that only those with higher pain severity had a decrease in social participation. Therefore, the finding of the severity of both neuropathic and nociceptive pain impacting on community reintegration in this current study is meaningful when considering reintegration into outcome‐based rehabilitation levels IV and V, where PWSCI are working towards participating in their community as well as potential employment.^58^

## STRENGTHS AND WEAKNESSES OF THE STUDY

This study allowed for analysis of both nociceptive and neuropathic pain experienced by people living with SCI, and its relationship to community reintegration. Furthermore, included participants were from a broad geographical area, with participants who had received rehabilitation at both public and private rehabilitation facilities, therefore allowing for more generalization of results. This study delimited the participants to only those with paraplegia which allowed influences of neurological fallout and weakness to not contribute to the shoulder pain experienced. The surgical procedures the five participants reported may have played a role in the WUSPI scores, and more information (such as success, recovery, etc.) may have given more context to the shoulder pain reported. However, according to the literature, there is a high prevalence of shoulder pain, but in this study, there was a small sample of participants with shoulder pain, which also may have influenced the results.

## CLINICAL IMPLICATIONS AND RECOMMENDATIONS

It is important that while trying to maximize PWSCI's independence and participation, we minimize potential shoulder pain. Strategies to decrease shoulder load may need to be implemented. We recommend that future shoulder pain research determine whether PWSCI were educated on shoulder pain prevention, and if they are applying prevention strategies, determine the efficacy of the pain prevention program. As shoulder pain severity was of particular importance concerning community reintegration, therapists must address shoulder pain, particularly self‐management strategies and education on shoulder care. Determining easing factors of pain and what self‐management strategies are used may help guide current in‐patient management. Neuropathic pain remains an important secondary health condition that should be managed adequately, as the severity of the neuropathic pain in this study was found to be linked with reduced community reintegration. Neuropathic pain should be assessed in relation to specific activities that pain interferes with, as people with neuropathic pain often continue to perform tasks despite their pain. Considering individual's specific needs, and their idea of integration and participation is therefore encouraged.

## CONCLUSION

This study highlights that pain is prevalent post‐SCI, and PWSCI participants continue with activities despite pain. However, a strong negative correlation was found between shoulder pain during car transfers and taking trips out of town. Furthermore, finding that the second‐most severe pain (mainly identified as neuropathic) and third‐most severe pain (P3) (mainly identified as shoulder pain) negatively correlated with moving around the community, and taking trips out of town and overall community reintegration respectively, makes the authors conclude that it is not the general presence of pain that influences community reintegration but the severity of pain. The type and severity of pain are important factors to consider to optimize the rehabilitation of a person back into their community.

## AUTHOR CONTRIBUTIONS

V.H and M.K.M were responsible for conceptualizing the study and its design. M.K.M collected the data as part of her umbrella PhD project and V.H. analyzed this data pertaining to community reintegration. Both authors contributed to interpretation of data, as well as writing of the manuscript.

## FUNDING INFORMATION

No funding was received for this work. The SANLiC agreement between University of the Witwatersrand and Wiley covered the Open Access costs.

## CONFLICT OF INTEREST STATEMENT

The authors declare that they have no financial or personal relationship(s) that may have inappropriately influenced them in writing this article.

## DISCLAIMER

The views and findings expressed in this manuscript are solely those of the authors and not an official position of the affiliated institutions. The authors are responsible for this article's results, findings, and content.

## Data Availability

Data sharing is not applicable to this article as no new data were created or analyzed in this study. Data sharing does not apply to this article as no new data were created in this study. The primary data analysed in this study can be made available from the University of Pretoria's physiotherapy department at a reasonable and ethical request.
